# Increasing health insurance enrolment in the informal economic sector

**DOI:** 10.7189/jogh.10.010329

**Published:** 2020-06

**Authors:** Adeola Ayodotun Onasanya

**Affiliations:** Social Policy Department, Nigerian Institute of Social and Economic Research, Ibadan, Nigeria

The Nigeria National health insurance scheme (NHIS) is a program that was designed and managed by the Federal ministry of Health to reduce the catastrophic out -of-pocket expenditure for health care in Nigeria. The NHIS aims to complement government efforts to support inadequate health budget allocation, reduce the burden of the rising costs of health care, as well as improve access to health care. The NHIS scheme was established in 1999 with the eventual operationalization in 2005. The scheme has different programmes. These include the formal economic health sector programme which covers the federal, state and local governments, and the organised private sector; the informal economic sector programme and the exemption group [1,2]. Despite the importance of the scheme to defraying health care costs and target of achieving universal health coverage by 2015, only about 3% of the population is covered by the scheme [1]. In addition, the formal economic sector scheme accounts for almost all of the total enrolled persons under the scheme [2].

Several factors have been attributed to why Nigeria has not met the 2015 target. One of the major reasons identified is poor public awareness with younger people having lower enrolment rates [1]. Other reasons for poor enrolment of health insurance include: normative ideas regarding governments role in full funding of the citizens’ health care, wrong beliefs about health insurance attracting ill-health, poor trust in governance, amongst others [1,3].

Since Nigeria has a large informal economic sector [2], efforts were made by the National council on health in 2015 to scale-up the scheme with the approval of state ownership of health insurance schemes as well as community-based health insurance. Although community-based health insurance is still in its infancy stage in Nigeria, there is evidence that the absence of communities in the co-creation, planning and implementation of the scheme can lead to low levels of health insurance enrolment [4]. In addition, lack of adequate information and sensitization of target participants has contributed to low health insurance enrolment [1]. As such, there is a need to develop strategies that involves key stakeholders and users in the creation, design and implementation of health communication packages for health insurance schemes. Moreover, targeting end users with adequate information and using target group demography in planning for and designing sensitization messages, can facilitate an increase in health insurance enrolment in Nigeria. One way to achieve this goal is to use principles from social network theory to identify ways to reach and involve end-users in health insurance enrolment. In addition, there is also a need to use the new trends in information and communication technology especially social media to gain the attention of target audience for the scheme.

## COMMUNICATING HEALTH INSURANCE INFORMATION

**Figure 1:**
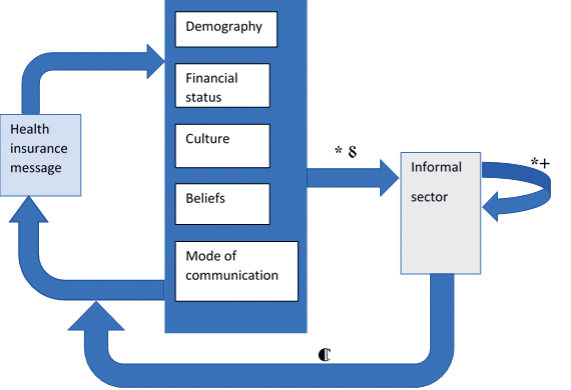
Proposed model of communicating health insurance to the informal economic sector. *social media, +word-of-mouth, ⸹mass media, ⸿evaluation.

To address this challenge of low health insurance uptake, it is critical to intensify public awareness by increasing conversation around health insurance through the use of media as well as designing communication packages on health insurance that is demography specific. For instance, information for market traders in the informal economic sector may vary from information for young persons in the private sector. One way this can be achieved is to use knowledge of the behaviour, norms and health care perspectives of population segment in designing health insurance information and communication model (**Figure 1**). Aligning health insurance information with beliefs, behaviour and social networks can increase enrolment in two ways. First, it fosters a sense of inclusiveness in that health insurance packages are fitted to the needs of end-users, which can increase the likelihood of health insurance enrolment. Second, the use of demography-specific information, as well as communication pathway, can increase conversations about health insurance within social networks leading to an increase in health insurance enrolment.

Although most Health Maintenance Organizations (HMOS) use traditional mass media in the form of radio and television jingles to pass along information, there is a need to pay attention to informal means of communication such as social media (including new media) and word-of-mouth (electronic and oral) to pass across health insurance information for several reasons. First, Nigeria has a large population of young people. An estimated 30% of the population are between ages 20-39 [5] and a substantial proportion of this young people would be in the informal economic sector. Since young people are less likely to be sick, pooling funds from a large proportion of this group can reduce health insurance premiums to achieve a cheaper and more affordable health care expenditure. Second, Nigeria has a large population of people, who use the internet and social media. Currently, about 59% (113.3 million) of Nigerians use mobile internet [6]. A large number of Nigerians also use social networks and messaging applications. For instance, 41% of the Nigerian population use Facebook and WhatsApp [7]. Out of these users, young people within the age range of 18-34 account for between 34%-80% of users on the major social media sites in Nigeria [8]. This implies that there is a high likelihood of attracting young people into taking up health insurance if HMOs target their social networks on social media in addition to other offline methods. It is counter-intuitive to the current thoughts that the informal economic sector may likely have low levels of education or access to technology. Lastly, oral word-of-mouth marketing is important in reaching the parts of the informal economic sector, which may have no formal economic education and or, are less likely to use social media. As such tapping into social networks of the different demographic and economic categories is likely to yield high gains in health insurance enrolment. Currently, there is little evidence of social network marketing of health insurance in Nigeria. Evidence from studies from the USA and Ghana on the use of social network concepts in disseminating health insurance information in both rural and urban communities using both electronic media and word-of-mouth models points to positive outcomes of undertaking social marketing for health insurance[9,10].

**Figure:**
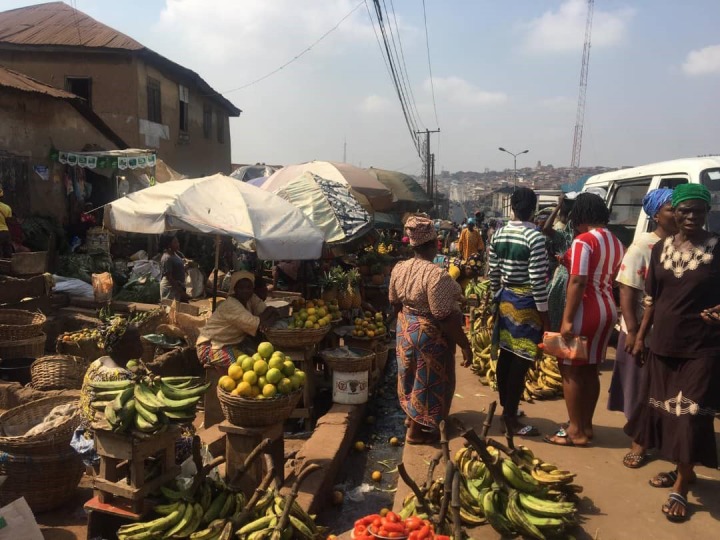
Photo: An open air market in Nigeria (from the author's collection, used with permission).

This impact of social network is not limited by geography as evidence suggest that the social media networks of a person can also influence health behavior [9]. Hence, the widespread use of social media is profoundly changing the way society and health care operates for several reasons. First, information that would hitherto have been delayed or unavailable can be available instantly. For instance, information about health care from social media and the Internet, reduced waiting times through the use of telemedicine. Second, finding a large number of networks with similar information can lend credence to the information.

## CONCLUSION

As stakeholders are increasing the scope of the NHIS in the informal economic sector, there is still much to be done in increasing health insurance coverage in Nigeria. It is therefore imperative that stakeholders should increase awareness of health insurance schemes through social networking. The successful enrolment on the health insurance scheme by a large portion of the population will involve leveraging on the use of both formal and informal means of communicating the benefits, as well as using demography specific communication patterns to showcase the importance of health insurance to the population. This can include the use of social media, leveraging on the social capital of media influencers, arts and storytelling through the entertainment industry as well as indigenous communication tools to pass across information on the benefits of health insurance. The effective use of communication pathways within social networks to increase awareness can also increase understanding of the health insurance concept, dispel negative beliefs associated with prepayment schemes, and increase the adoption of health insurance as a norm within communities.
